# Supplementary material for: Comparison of gut microbiome in the Chinese mud snail (Cipangopaludina chinensis) and the invasive golden apple snail (Pomacea canaliculata)
Source: PeerJ. 2022 Apr 5;10:e13245. doi: 10.7717/peerj.13245 (PMC8992660; doi:10.7717/peerj.13245)
Supplement: Supplemental Information 1 [file peerj-10-13245-s001.docx]

**An insight into the role of gut microbiota in successful invasion of golden apple snail (*Pomacea canaliculata*)**

Zi-Hao Zhou^1, 2, 4^, Hong-Yin Wu^4^, Ding-Hong Li^4^, Wen-Long Zeng^4^, Jin-Long Huang^1, 2, 3, 4 *^, Zheng-Jun Wu^1, 2, 4, *^

^1^ Key Laboratory of Ecology of Rare and Endangered Species and Environmental Protection (Guangxi Normal University), Ministry of Education, Guilin, Guangxi, China

^2^ Guangxi Key Laboratory of Rare and Endangered Animal Ecology, Guangxi Normal University, Guilin, Guangxi, China

^3^ Guangxi Key Laboratory of Landscape Resources Conservation and Sustainable Utilization in Lijiang River BasinInstitute for Sustainable Development and Innovation, Guangxi Normal University, Guilin, Guangxi, China

^4^ College of Life Sciences, Guangxi Normal University, Guilin, Guangxi, China

*Corresponding author:

Jin-Long Huang ([jl_huang@163.com](mailto:jl_huang@163.com)), Zheng-Jun Wu (wu_zhengjun@aliyun.com)

Address: College of Life Sciences, Guangxi Normal University, Guilin, 541006, China.

**Figure S1** Rarefaction Curve based on Shannon index. The blue lines indicate the rarefaction curve of PC groups (PC 1-4), and red lines indicate to CC groups (CC 1-4). Rarefaction curves of samples reached asymptote, indicating that sequencing depth basically covered all species in the samples.


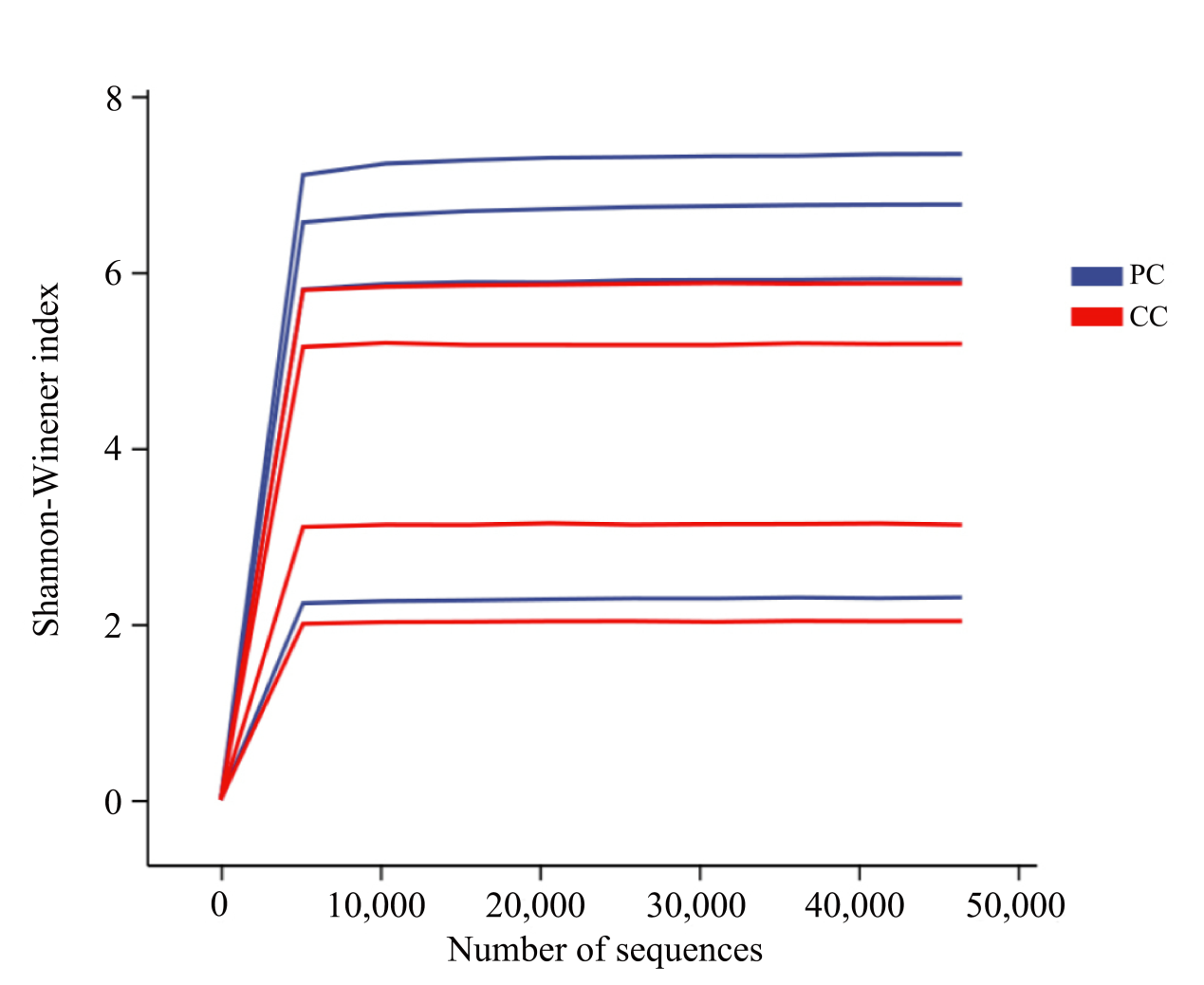


**Figure S2** The content of operational taxonomic units (OTUs) and different bacterial taxonomic units of each sample. The y-axis represents different samples in PC and CC groups, x-axis represents the total annotated OUT numbers. Different colors represent different classification level


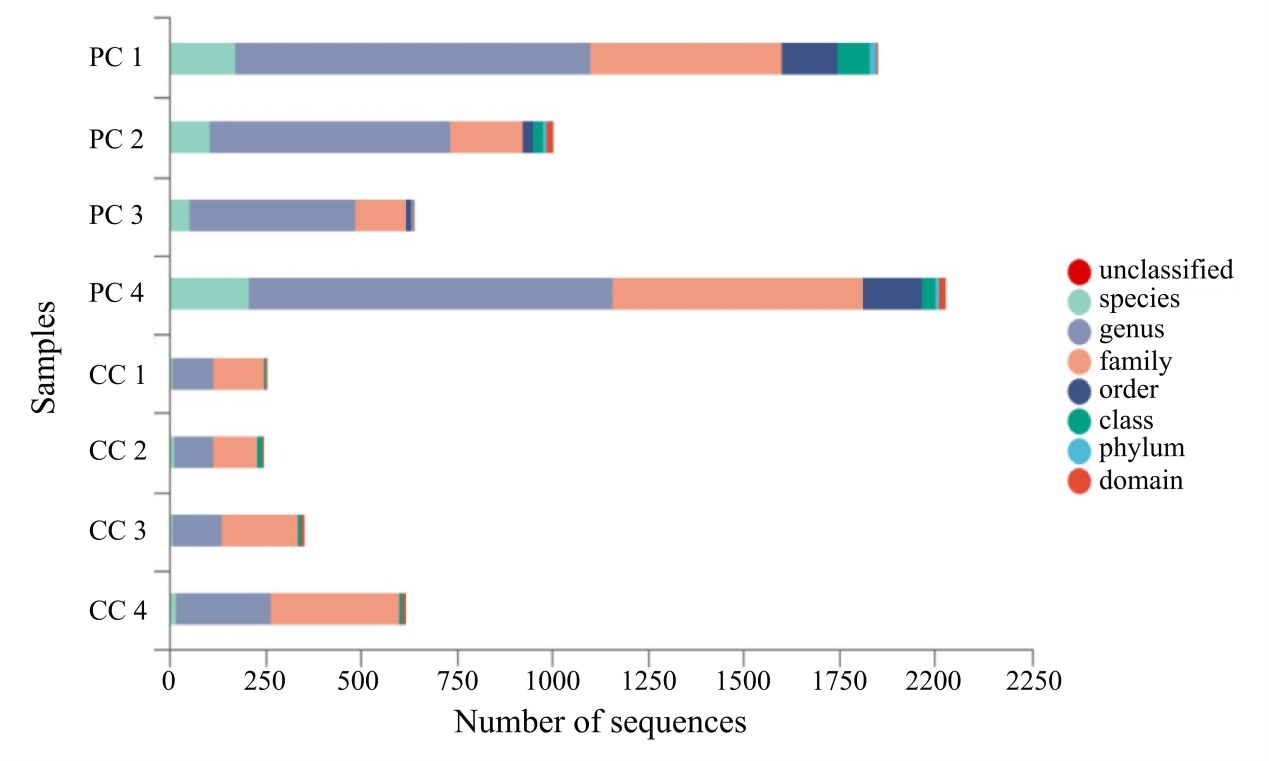


**Figure S3** Venn diagram of shared and unique OTUs among the PC and CC groups.


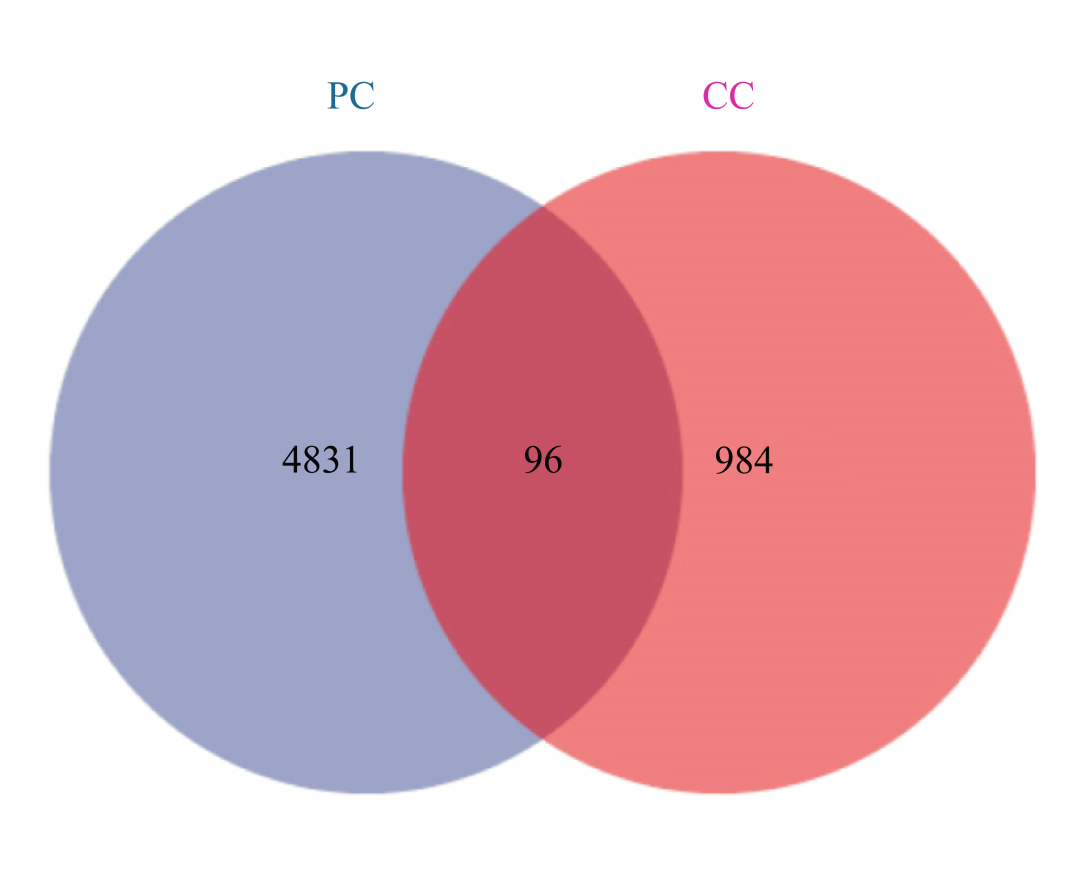


**Figure S4** PCoA analysis based on (A) unweighted and (B) weighted Unifrac distances of gut microbiome on OTU level. Part of the dots have overlapped (A and B) rather than samples lacked.


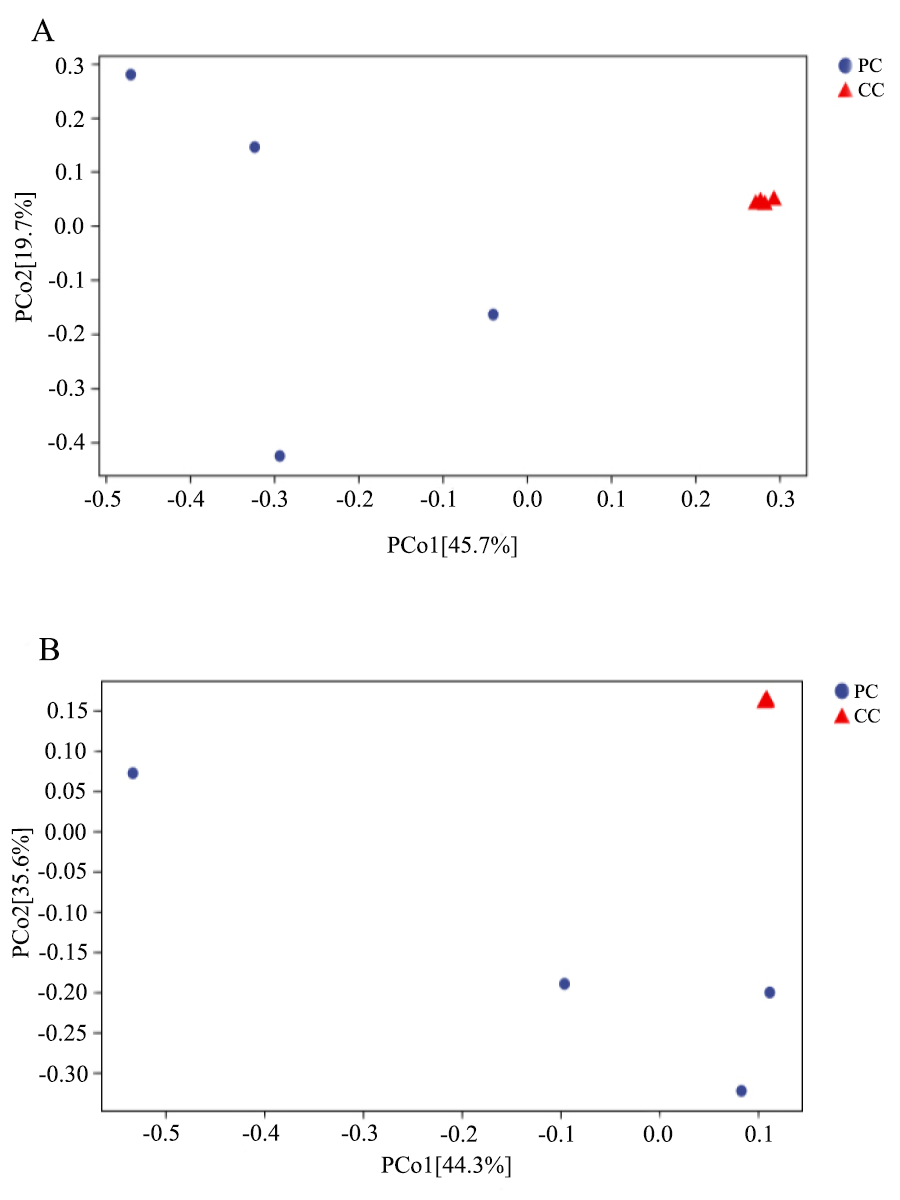


**Figure S5** The linear discriminant analysis (LDA) of two groups with the LDA >2. The y-axis represents the significantly different OTUs between PC and CC groups. The x-axis represents the LDA Score (log10). The length of histograms represents the significantly different level of OTUs.


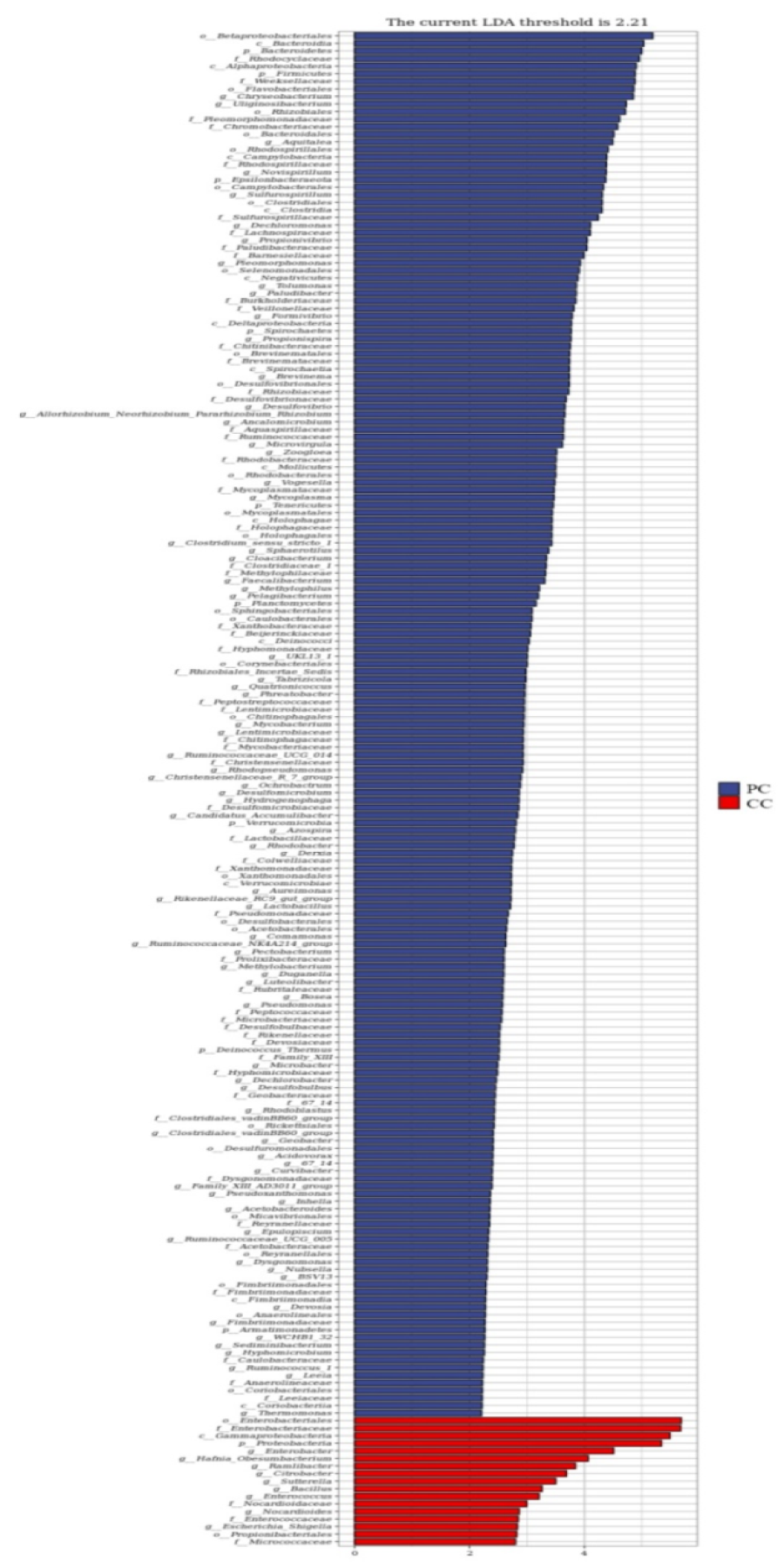


**Figure S6** The prediction of two groups in KEGG database in different annotation abundance. The top 20 KEGG pathways (level 2) were concluded to KEGG pathways (level 1) on the left side.


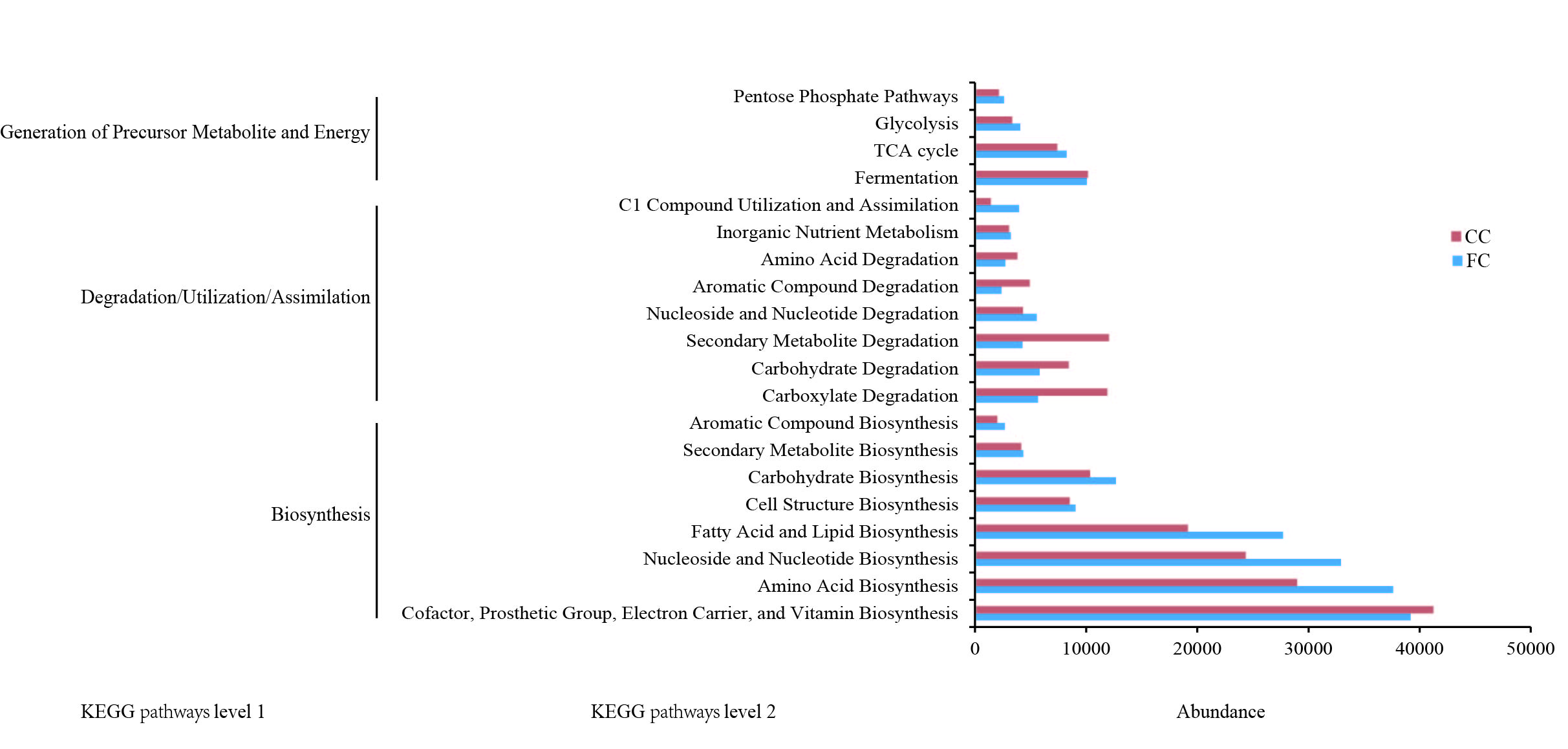


Table S1 The relative abundance of top 10 microbiota of each group at phylum level.

| Phylum | PC 1 (%) | PC 2 (%) | PC 3 (%) | PC 4 (%) | PC group  (Mean ± sd) (%) | CC 1 (%) | CC 2 (%) | CC 3 (%) | CC 4 (%) | CC group  (Mean ± sd) (%) |
| --- | --- | --- | --- | --- | --- | --- | --- | --- | --- | --- |
| Proteobacteria | 78.30 | 64.40 | 8.10 | 64.40 | 53.78 ± 15.59 | 98.40 | 98.90 | 99.40 | 99.10 | 98.94 ± 0.20 |
| Bacteroidetes | 7.50 | 9.30 | 56.40 | 19.70 | 23.19 ± 11.38 | 1.00 | 0.4 | 0.06 | 0.08 | 0.37 ± 0.21 |
| Firmicutes | 2.80 | 12.30 | 35.4 | 13.50 | 16.00 ± 6.91 | 0.3 | 0.7 | 0.4 | 0.6 | 0.49 ± 0.08 |
| Epsilonbacteraeota | 10.00 | 7.10 | 0.01 | 0.03 | 4.28 ± 2.53 | 9.65E-3 | 4.00E-3 | 0 | 8.40E-3 | 0.01 ± 0.00 |
| Spirochaetes | 0.10 | 3.80 | 7.48E-3 | 0.09 | 1.00 ± 0.92 | 0 | 0 | 0 | 0 | 0 |
| Acidobacteria | 1.10 | 0.20 | 0 | 0.90 | 0.56 ± 0.27 | 2.41E-3 | 0 | 0 | 2.80E-03 | 0 |
| Tenericutes | 6.13E-03 | 2.00 | 0 | 0.05 | 0.52 ± 0.50 | 0 | 0 | 0 | 0 | 0 |
| Actinobacteria | 0.10 | 0.30 | 0.10 | 0.80 | 0.33 ± 0.17 | 0.20 | 0.08 | 0.10 | 0.2 | 0.14 ± 0.03 |
| Verrucomicrobia | 0.10 | 0.04 | 11.2E-3 | 0.3 | 0.09 ± 0.06 | 4.82E-3 | 0 | 0 | 4.20E-3 | 0 |
| Fibrobacteres | 0 | 0. 20 | 0 | 0 | 0.06 ± 0.06 | 0 | 0 | 0 | 0 | 0 |
| other | 0.05 | 0.39 | 0.03 | 0.27 | 0.19 ± 0.09 | 0.06 | 0.03 | 0.03 | 0.07 | 0.05 ± 0.01 |

Table S2 The relative abundance of top 10 microbiota of each group at genus level

| Genus | PC 1 (%) | PC 2 (%) | PC 3 (%) | PC 4 (%) | PC group  (Mean ± sd) (%) | CC 1 (%) | CC 2 (%) | CC 3 (%) | CC 4 (%) | CC group  (Mean ± sd) (%) |
| --- | --- | --- | --- | --- | --- | --- | --- | --- | --- | --- |
| Chryseobacterium | 0. 5 | 2.80 | 54.30 | 0.90 | 14.62 ± 13.24 | 0 | 0 | 0 | 0 | 0 |
| Lactococcus | 0. 1 | 4.70 | 34.10 | 0.2 | 9.78 ± 8.18 | 0.05 | 0.50 | 0.20 | 0.40 | 0.26 ± 0.10 |
| Uliginosibacterium | 13.10 | 25.70 | 2.14E-3 | 0.07 | 9.73 ± 6.16 | 0 | 0 | 0 | 0 | 0 |
| Enterobacter | 0.02 | 0.03 | 0.03 | 0.08 | 0.04 ± 0.01 | 4.40 | 1.40 | 11.40 | 9.80 | 6.73 ± 2.31 |
| Aquitalea | 14.80 | 3.70 | 0.02 | 6.10 | 6.13 ± 3.13 | 0 | 0 | 0 | 0 | 0 |
| Novispirillum | 7.40 | 9.00 | 0.02 | 0 | 4.11 ± 2.39 | 0 | 0 | 0 | 0 | 0 |
| Sulfurospirillum | 9.40 | 4.50 | 0.01 | 0.03 | 3.50 ± 2.24 | 0 | 0 | 0 | 0 | 0 |
| Bacteroides | 0.1 | 0.2 | 0.4 | 8.00 | 2.17 ± 1.95 | 0.91 | 0.36 | 0.31 | 8.40E-3 | 0.33 ± 0.21 |
| Hafnia-Obesumbacterium | 0 | 0 | 0 | 0 | 0 | 0 | 0.90 | 0.08 | 9.00 | 2.49 ± 2.13 |
| Dechloromonas | 5.50 | 1.70 | 0.03 | 2.60 | 2.46 ± 1.15 | 0 | 0 | 0 | 0 | 0 |
| other | 49.04 | 47.73 | 11.08 | 81.99 | 47.46 ± 14.49 | 94.63 | 96.88 | 88.38 | 80.86 | 90.19 ± 3.59 |
